# Leptandrin

**Published:** 1907-10

**Authors:** James Burke

**Affiliations:** Manitowoc, Wis.


### LEPTANDRIN.

By James Burke, M. D., Manitowoc, Wis.

Leptandrin is a resin derievd from Leptandra Virginica, or Culver's root. The resin is a complexity; it contains a proteid allied to the protoplasmic and enzymic proteid of the liver cells and the digestive ferment glands of the stomach and intestines; in the first stage of aberration of these cells, early in its course, Leptandrin alone will suffice to set them right, but when the acute fever process has been allowed to progress to the formation of toxins in the walls of the stomach and intestines, the lumen of the bowel must be cleared of the fermenting contents, and subsequently the accumulated toxins in the stomach and intestines, must be neutralized by the indicated use of emetine, piglandin, iridin, chionanthin, colocynthin, or podophyllin, before the virtues of leptandrin can be brought out in neutralizing liver cell toxins and otherwise beneficially modifying the ferment producing cells of the stomach, liver and intestines. Preparations of the fresh root of leptandra contain a volatile principle, not ye isolated, evidently a strong proteid poison, which, when taken by the stomach, chemically attacks its allied substances in the construction of the stomach and bowels, to round itself out to become an excretory product, that is manifested in dangerous emesis, catharsis, dysentery, vertigo and foetal miscarriage. The crude drug must be dried for a sufficient time to allow this principle to be dissipated, before the drug is fit for extraction of its benevolent medicinal qualities.

When large doses of leptandrin are given by the stomach; or when smaller doses are given, which the digestion of the stomach and intestines cannot properly harmonize, for the better therapeutic action of the drug, the ingested drug proceeds to satisfy its chemical wants on the mucous membrane of the stomach and intestines, with the result of producing purgation. There are better laxatives and purgatives. Leptandrin does its best therapeutic work just short of the laxation of the bowels.

Thus used, in stimulating to proper action, the liver, the glands of the stomach and intestines, leptandrin is useful in intestinal atony; indigestion with deficient secretion by adding podophyllin; combined with hydrastine in gastric atony; dyspepsia with frontal headache, yellow tongue, nausea and jaundice, combined with jugulandin, dioscorein and aloin in small repeated doses; for diarrhoea with passage of undigested food, inactive liver, dull abdominal pain and clayey stools, combined with aconitine, juglandin and colocynthin; in diarrhoea of dentition, combined with hyoscyamine and sulpho-carbolate of lime; jaundice with tender liver in adults, add aconitine, hyoscyamine and strychnine; in malaria after the chills are broken, it is essentially assisted by small, oft-repeated doses of aconitine, digitaline or strychnine, or a combination of the three to procure a grade of circulatory pressure, and distribution of the blood and fluids, approaching to the normal standard.

In dropsy and ascites, without any serious organic lesion of the liver, heart or kidneys, leptandrin is not useful until the pressure of the fluid accumulation is relieved by draining off a portion of the fluid; emptying the bowels of their fermenting contents; subsequently thereto, preventing further fermentation of the bowel contents by requisite doses of the sulphocarbolates, acetozone, alphozone or some other suitable antiferment; next, proceed to neutralize the stomachic and intestinal proteid toxins by the use of the cognate proteid substances, emetine, hydrastine, juglandin, iridin, chionanthin, colocynthin or podophyllin as indicated. This done, then, the leptandrin, boldine and dioscorein administered must have a better therapeutic effect. Depleting the body of fluids through the bowel route, through hypercatharsis, but debilitates the patient still more; and besides, the utmost consideration must be given to rounding out the toxins, which are normally excreted by the kidneys. All other toxins must receive like consideration. Dropsy is generally a systemic rather than a regional ailment; even in cardiac insufficiency, if proper feeding and necessary attention is given to elimination, the cardiopathy will furnish a minimum of annoyance.

